# Hederagenin Promotes Sorafenib Sensitivity in Hepatocellular Carcinoma Through Suppressing SLC7A11 Expression and Inducing Ferroptosis

**DOI:** 10.1002/fsn3.71873

**Published:** 2026-05-22

**Authors:** Hai‐mei Jiang, Yu‐wan Zhou, Jian‐yu Zhang, Fu‐zhen Pan, Jin‐Fang Zhang, Ya‐ge Zhang

**Affiliations:** ^1^ Cancer Center Shenzhen Hospital (Futian) of Guangzhou University of Chinese Medicine Shenzhen China; ^2^ Research Institute Shenzhen Hospital (Futian) of Guangzhou University of Chinese Medicine Shenzhen China; ^3^ The Sixth Clinical Medical College Guangzhou University of Chinese Medicine Shenzhen China; ^4^ College of Chinese Medicine Macau University of Science and Technology Macau China

**Keywords:** ferroptosis, hederagenin, hepatocellular carcinoma, sorafenib

## Abstract

Sorafenib (SOR) significantly improves the survival rate for patients with advanced hepatocellular carcinoma (HCC). Nevertheless, the clinical efficacy is restricted by the development of acquired resistance. Therefore, enhancing susceptibility of HCC cells to SOR is of critical importance for patients with advanced disease. Traditional Chinese medicine (TCM) possesses considerable potential to overcome SOR resistance. Hederagenin (HED), a bioactive triterpenoid molecule with potent antitumor activity, has been reported to markedly reduce chemoresistance to various anticancer drugs in cancer cells. In the present study, we observed that HED synergistically potentiated the anticancer activity of SOR in HCC cells, reducing its IC50 by more than 50%. Further investigations revealed that HED increased SOR‐induced ferroptosis, and inhibition of ferroptosis clearly abrogated this synergistic effect. Mechanistically, SLC7A11, a key regulator of ferroptosis, was identified as a potential target of HED in HCC. And our data further revealed the pronounced upregulation of SLC7A11 in human HCC tissues, whereas HED treatment significantly downregulated its expression, suggesting that SLC7A11 might be a principal molecular target of HED in SOR‐mediated ferroptosis. Furthermore, loss‐ and gain‐of‐function experiments confirmed that SLC7A11 plays an essential role in mediating HED‐enhanced SOR‐induced ferroptosis. In summary, our results demonstrated that HED synergistically promoted the anti‐cancer effects of SOR on HCC cells by suppressing SLC7A11 expression, thereby triggering ferroptosis. These results suggest that HED represents a promising strategy to overcome SOR resistance and offers a viable therapeutic approach to improve SOR efficacy in patients with resistant HCC.

## Graphical abstract

This study combining network pharmacology with molecular docking further confirmed via in vitro experiments that HED potentiates the anti‐tumor effect of sorafenib in HCC cells through the downregulation of SLC7A11 expression and the induction of ferroptosis.

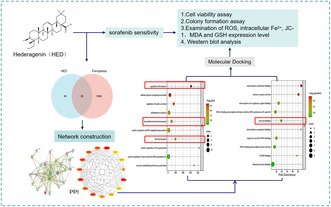

## 1 Introduction

Hepatocellular carcinoma (HCC) represents over 50% of cancer diagnoses in China and ranks as the third most common cause of cancer‐related fatalities globally (Chen et al. 2019). This alarming statistic underscores the urgency for effective prevention and treatment to combat this aggressive form of cancer. Although treatment options such as surgical resection, liver transplantation, and transcatheter arterial chemoembolization initially show therapeutic effectiveness, the long‐term results are often insufficient because of elevated rates of tumor recurrence and mortality, which markedly restrict the success of surgical procedures (Anwanwan et al. 2020). Sorafenib, which inhibits multiple kinases, is utilized as the primary systemic treatment for advanced HCC. Nevertheless, it only provides clinical advantages to approximately 30% of patients, while most experience drug resistance within 6 months of starting treatment (Zhu et al. 2017). Thus, novel therapeutic strategies that can overcome SOR resistance while maintaining favorable safety profiles are urgently needed.

Ferroptosis, a distinct, controlled, iron‐dependent cell death, is marked by a buildup of intracellular iron alongside an excess of reactive oxygen species (ROS). This mechanism results in heightened oxidative stress and lipid peroxidation (Jiang et al. 2021). Notably, this particular form of cell death has been involved in resistance against cancer therapies (Zhang, Liu, et al. 2022). According to a previous study, sorafenib promotes ferroptosis in HCC by blocking the cystine/glutamate antiporter system Xc^−^ (xCT/SLC7A11 complex), resulting in iron‐dependent cell death (Shi et al. 2025). The system Xc−, which functions as a cystine‐glutamate antiporter, is well‐known for its critical role in regulating ferroptosis and is encoded by the solute carrier family 7, membrane 11 (SLC7A11) gene (Jiang et al. 2021). Therefore, one possible strategy for reversing sorafenib resistance in HCC might be to reduce ferroptotic activity.

Traditional Chinese herbal medicine (TCM) offers novel therapeutic potential for overcoming resistance to sorafenib in HCC. Many compounds derived from TCM provide great promise to resolve this challenge. Hederagenin (HED), a bioactive triterpenoid compound, exhibits potent antitumor activity and has been demonstrated to significantly inhibit malignant tumor progression (Zhang et al. 2024). Notably, growing evidence suggests that HED increases cancer cells' susceptibility to several anticancer medications. Specifically, it has been noted that HED enhances the response of lung cancer cells to both paclitaxel and cisplatin (Wang et al. 2020; Sun et al. 2025), while also effectively reversing oxaliplatin resistance in gastric cancer cells (Tang et al. 2024). Furthermore, research has demonstrated that the compound HED can activate a ferroptosis pathway, which relies on the presence and activity of the protein CHAC1, and induce lung cancer cell death (Lu, Guo, et al. 2024). Nonetheless, it remains unclear whether HCC cells can be sensitized to sorafenib and the role that ferroptosis may play in this process. In this study, we demonstrated that HED facilitated ferroptosis induced by sorafenib and overcame resistance to the drug in HCC, suggesting a novel synergistic approach for treating HCC through the combination of HED and sorafenib.

## 2 Materials and Methods

### 2.1 Reagents and Antibodies

This study was carried out at the Shenzhen Hospital (Futian) of the Research Institute of Guangzhou University of Chinese Medicine, located in Shenzhen, China. Reagents used in the present study included Sorafenib (HY‐10201), hederagenin (HY‐N0256), and a variety of inhibitors, including Z‐VAD (HY‐164388) for apoptosis, Chloroquine (HY‐17589A) for autophagy, and Ferrostatin‐1 (Fer‐1, HY‐100579) for ferroptosis, all purchased from MedChemExpress (China). Antibodies for SLC7A11 (T57046), GPX4 (T56959), and β‐actin (P30002) were purchased from Abmart (Shanghai, China).

### 2.2 Cell Culture

The Huh7 and HepG2 cell lines were sourced from the ATCC (Manassas, VA, USA) and were grown in DMEM that was enriched with 10% FBS. They were kept at 37°C within a 5% CO_2_ environment.

### 2.3 Cell Viability Assay

The CCK‐8 assay, a widely accepted method for determining cell health and proliferation, was employed to evaluate cell viability. In this study, various concentrations of HED and/or SOR were administered to the cultures. After 24 h of incubation, detection was performed following the CCK‐8 manual. Every experiment was performed three times to guarantee the consistency and repeatability of the findings (Shi et al. 2022).

### 2.4 Colony Formation Assay

Huh7 and HepG2 cell lines were seeded (200 cells/well) in 6‐well plates, followed by culturing in a medium enriched with either 20 μM HED, 5 μM SOR, or a combination of both treatments (20 μM HED and 5 μM SOR) for a period of 2 weeks. The colonies underwent a rinsing process with PBS to eliminate any residual substances. Subsequently, the colonies were fixed in 4% formaldehyde and then stained with a 0.5% crystal violet solution for 30 min to enable effective visualization. Microscopic images of the stained colonies were then captured for accurate observation, and the colonies were precisely counted for subsequent analysis (Jiang et al. 2025).

### 2.5 Examination of ROS, Intracellular Fe^2+^, Mitochondrial Membrane Potential, MDA and GSH Expression Level

The S0033S Reactive Oxygen Species Assay Kit (Beyotime, Shanghai, China) was utilized for ROS quantification. To assess intracellular Fe^2+^ concentrations, FerroOrange staining (F374, DOJINDO, Shanghai, China) was employed. Mitochondrial membrane potential was assessed using the JC‐1 kit (Accurate Biology, China). To assess glutathione (GSH) and malondialdehyde (MDA) expression levels, a lipid peroxidation assay kit (DOJINDO, Shanghai, China) and a reduced GSH assay kit (NJJCBIO, China) were utilized, respectively. All experimental procedures were performed in strict accordance with the manufacturers' protocols.

### 2.6 Targets Prediction

The TCMSP database (https://www.tcmsp‐e.com/) and the Swiss Target Prediction database (http://swisstargetprediction.ch/) were utilized to predict the possible targets of HED (Feng et al. 2021). Targets associated with ferroptosis were identified using the GeneCards resource (https://www.genecards.org/) and the FerrDb database (http://www.zhounan.org/ferrdb/current/) (Deng et al. 2024). An online program (https://www.bioinformatics.com.cn) was utilized to create a Venn diagram. Furthermore, a protein–protein interaction (PPI) network, which was then imported into Cytoscape software (v3.9.1), was developed using the STRING database (https://cn.string‐db.org/). The top 15 hub genes were determined, applying the degree algorithm through the CytoHubba plugin. Gene Ontology (GO) analysis, which encompassed biological processes (BP), molecular functions (MF), and cellular components (CC), was carried out using the DAVID database (https://davidbioinformatics.nih.gov/). Visualization was performed on the aforementioned platform (Liu et al. 2024). SLC7A11 expression levels in HCC were examined utilizing the UALCAN platform (http://ualcan.path.uab.edu/) with data from The Cancer Genome Atlas (TCGA). Finally, a survival analysis for SLC7A11 in HCC individuals was conducted utilizing the Kaplan–Meier Plotter database (http://kmplot.com) (Chandrashekar et al. 2022).

### 2.7 Molecular Docking

The three‐dimensional (3D) configurations of the target proteins were generated from the RCSB Protein Data Bank (https://www.rcsb.org/). All water molecules and associated ligands were eliminated from the protein configurations using PyMOL (The PyMOL Molecular Graphics System, Version 2.5.1, Schrödinger LLC) to set them up for docking simulations. The 2D chemical structure of HED was accessed from the PubChem database (https://pubchem.ncbi.nlm.nih.gov/). Subsequently, the modified protein structures and the HED ligand molecule were uploaded to the CB‐Dock web server (http://clab.labshare.cn/cb‐dock/php/index.php) for the purpose of molecular docking analysis (Lu et al. 2025).

### 2.8 Cell Transfection

The SLC7A11 overexpression plasmid was constructed as described previously (Shi et al. 2025). The siRNA targeting SLC7A11 was purchased from GenePharma (Shanghai, China), and its sequence is provided in Table S1. Transfection into cells was performed using Lipofectamine 3000 (Invitrogen, USA) according to the manufacturer's instructions.

### 2.9 Western Blot Analysis

The process of protein extraction utilized RIPA lysis buffer (Beyotime, Shanghai, China), and then the protein quantification was conducted with a BCA protein assay kit (Thermo Fisher Scientific, USA). Equal amounts of protein samples were separated by 10% SDS‐PAGE gel electrophoresis and subsequently transferred to PVDF membranes (SLGV004SL‐1, Millipore, Massachusetts, USA). After a one‐hour blocking with 5% skim milk at room temperature, the membranes were treated with primary antibodies overnight at 4°C: β‐actin (1:2000), GPX4 (1:1000), and SLC7A11 (1:1000) (both from Abmart, Shanghai, China). The membranes were then treated with HRP‐conjugated secondary antibodies (Merck Millipore; diluted 1:5000) at room temperature for 1 h. The visualization of protein bands was achieved using enhanced chemiluminescence detection reagents (Millipore, MA, USA), with a chemiluminescence imaging system (Fluor Chem R, USA). Band intensity was quantitatively analyzed with ImageJ software.

### 2.10 Statistical Analyses

Statistical evaluations were conducted utilizing GraphPad Prism 9. The findings are shown as mean ± standard deviation (SD). To compare multiple groups, least significant difference tests were employed subsequent to a one‐way ANOVA to assess variations, with statistical significance set at *p* < 0.05 (*p < 0.05; **p < 0.01; ***p < 0.001).

## 3 Results

### 3.1 HED Significantly Promoted Sorafenib Sensitivity

**FIGURE 1:**
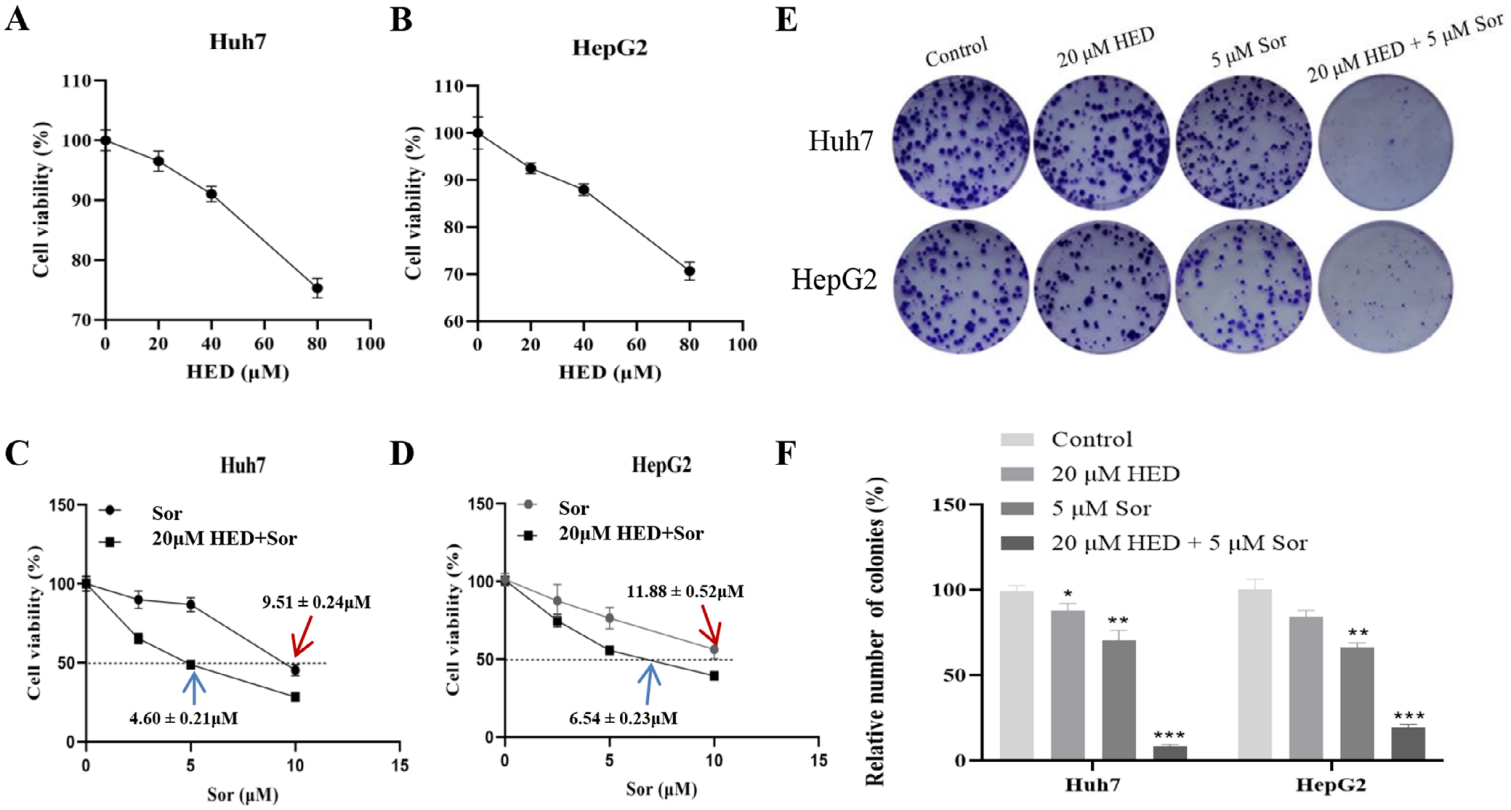
HED significantly promoted sorafenib sensitivity in HCC cells. (A, B) Viability of Huh7 (A) and HepG2 (B) cells after treatment with various concentrations of HED for 48 h, as assessed by the CCK8 assay. (C, D) Following treatment with 20 μM HED, cell viability was evaluated, and the IC_50_ value of SOR was determined. (E, F) The effects of the combination of HED and SOR on colony formation were analyzed. Data were presented as means ± SD (*n* = 3). *p < 0.05; **p < 0.01; ***p < 0.001, versus 5 μM Sor alone.

To examine the influence of HED on sorafenib resistance in HCC, the cytotoxic effects of HED on Huh7 and HepG2 cells were initially assessed. The results illustrated in Figure 1A,B revealed that HED reduced cell viability in both Huh7 and HepG2 cells in a dose‐dependent manner (Figure 1A,B). Considering that 20 μM HED demonstrated minimal cytotoxicity towards HCC cells, this concentration was chosen for subsequent investigations. Functional experiments indicated that co‐treatment with 5 μM sorafenib and HED led to a reduction in cell viability and colony formation (Figure 1C–F). Our findings also indicated that treatment with 20 μM HED for 24 h decreased the IC50 of sorafenib from 9.51 ± 0.24 to 4.60 ± 0.21 μM in Huh7 cells, and from 11.88 ± 0.52 to 6.54 ± 0.23 μM in HepG2 cells, thereby indicating that HED significantly improved sorafenib sensitivity in HCC cells. We also observed this phenomenon at 48 and 72 h (Figure S1A–D).

### 3.2 Inhibition of Ferroptosis Reversed the Synergic Effects Between HED and Sorafenib

**FIGURE 2:**
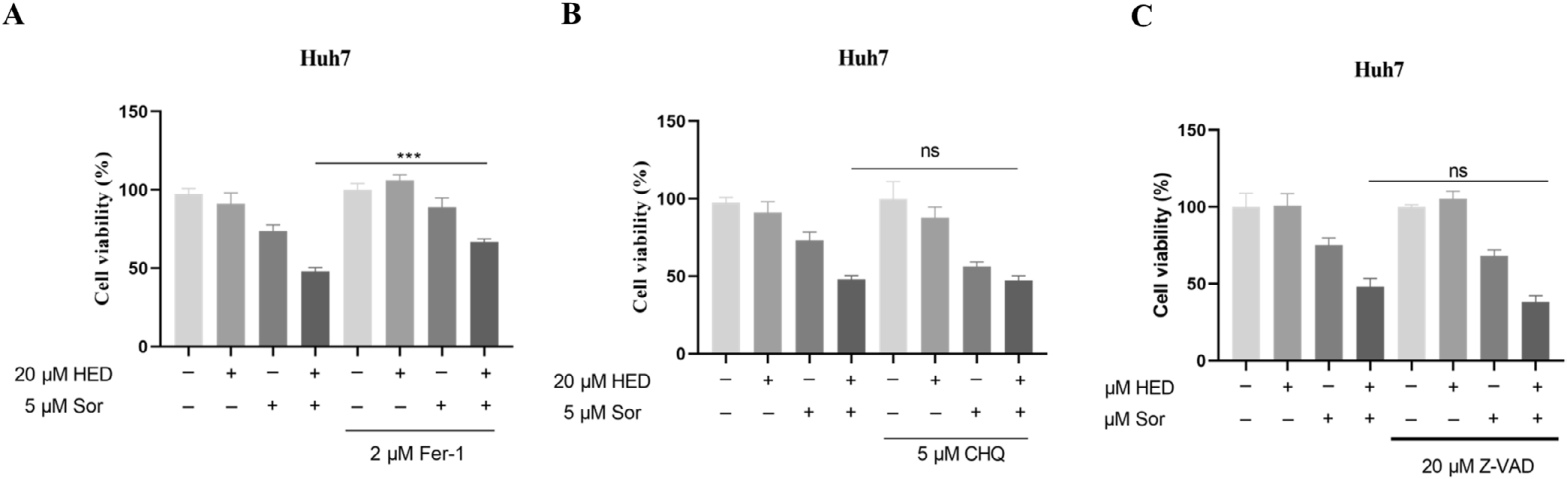
Inhibition of ferroptosis reversed the synergic effect between HED and SOR. Relative vitality of Huh7 cells after 24‐h treatment with HED and SOR, in the presence or absence of 2 μM Fer‐1 (A), 20 μM CHQ (B), 10 μM Z‐VAD (C), or without these drugs. Data were presented as means ± SD (*n* = 3). ***p < 0.001, versus 5 μM Sor alone.

Ferroptosis, characterized as a new cellular death, is triggered by sorafenib in HCC cells. To examine whether ferroptosis contributes to the sorafenib resistance mediated by HED, a rescue experiment was undertaken utilizing the ferroptosis inhibitor (Fer‐1), the autophagy inhibitor (CHQ), or the apoptosis inhibitor (Z‐VAD), respectively. The results indicated that the survival rate of Huh7 cells exposed to both HED and sorafenib was significantly increased when treated with 2 μM Fer‐1 (Figure 2A). On the other hand, there were no notable rescue results when the autophagy inhibitor CHQ or the apoptosis inhibitor Z‐VAD was applied to co‐treated cells (Figure 2B,C). These results suggest that HED improves the sensitivity of Huh7 cells to sorafenib mainly through inducing ferroptosis.

### 3.3 HED Promoted SOR‐Induced Ferroptosis

**FIGURE 3:**
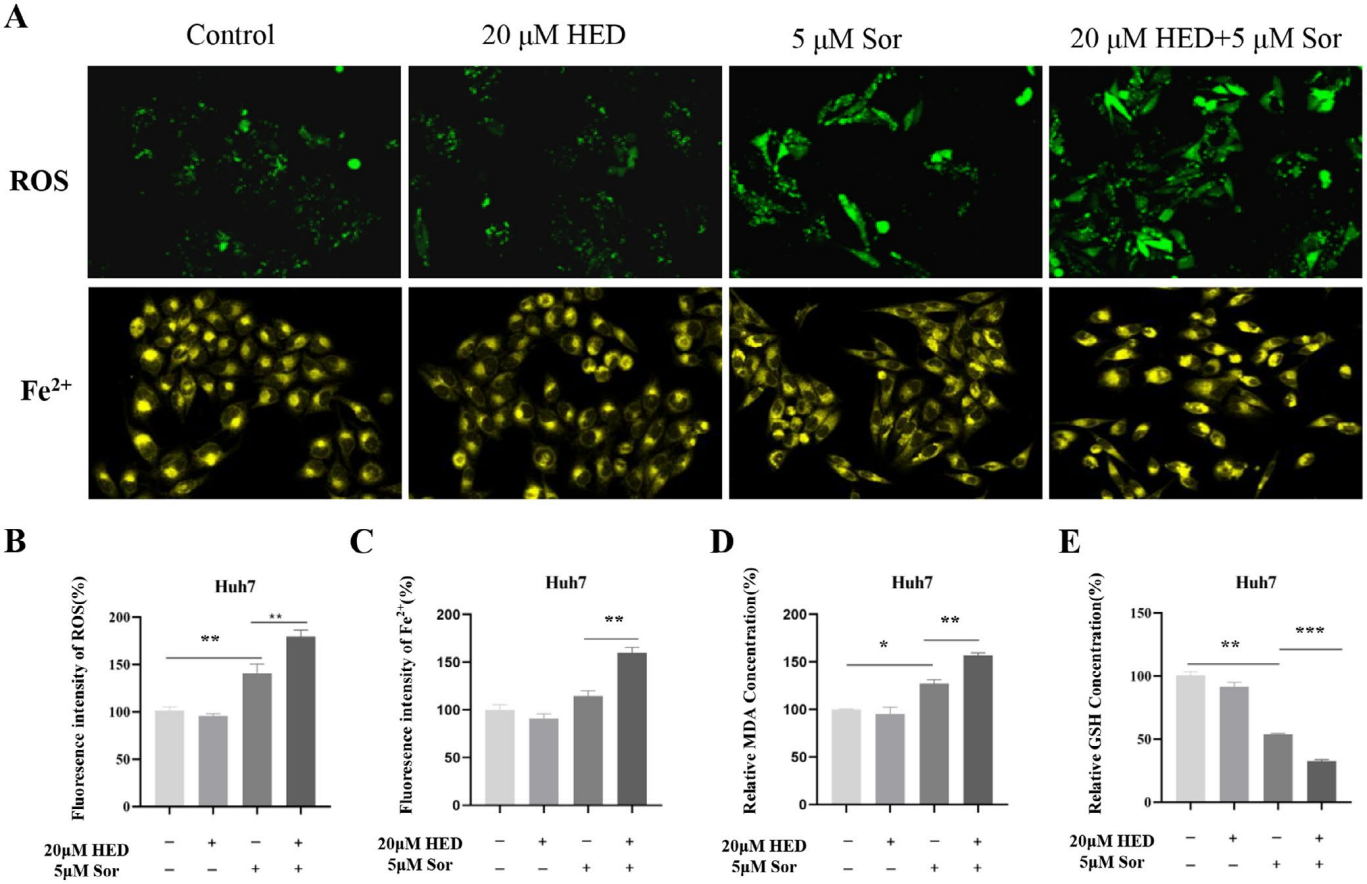
HED enhanced SOR‐induced ferroptosis in HCC cells. (A–C) Representative immunofluorescence images of ROS and Fe^2+^ (A) and quantification of fluorescence signals (B, C). (D, E) MDA and GSH levels were measured in Huh7 cells subjected to co‐treatment with HED and SOR. Data were presented as means ± SD (*n* = 3). *p < 0.05; **p < 0.01; ***p < 0.001, versus 5 μM Sor alone.

To further validate the role of HED in reducing sorafenib resistance, key markers associated with ferroptosis, including GSH, MDA, and the intracellular levels of iron and lipid ROS, were examined. An increase in fluorescence intensity for both ROS and Fe^2+^ was observed in the co‐treated cells (Figure 3A–C). Given MDA accumulation and GSH depletion are important markers of ferroptosis, we analyzed their expression levels in HCC cells. Comparing with the group only with SOR treatment, HCC cells with co‐treatment exhibited lower levels of GSH (Figure 3D) and elevated levels of MDA (Figure 3E). These findings indicate that HED improves the sensitivity to sorafenib by triggering ferroptosis in HCC.

### 3.4 The Downstream Targets of HED in Reversing SOR Resistance

**FIGURE 4:**
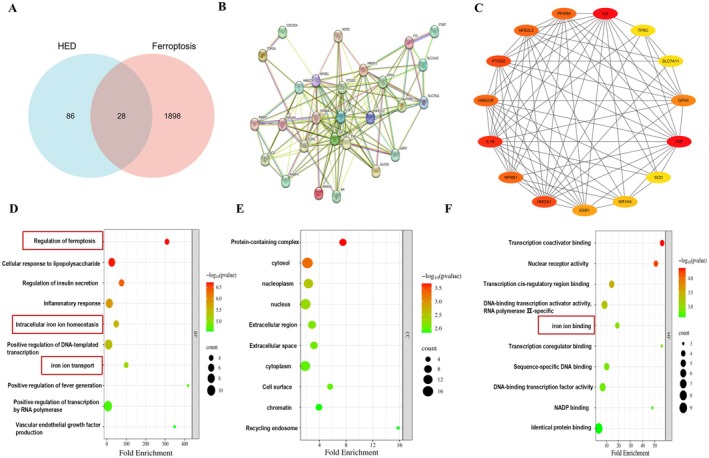
Downstream targets of HED were predicted by bioinformatics analysis. (A, B) Venn diagram alongside the PPI network illustrating the shared common targets between HED and ferroptosis. (C) Top 15 hub targets identified by Hubba in Cytoscape from the shared candidates. (D–F) GO enrichment analysis of these common targets, categorized into biological process (BP), cellular component (CC), and molecular function (MF).

To elucidate the fundamental mechanism underlying HED‐mediated SOR resistance, we performed a bioinformatics analysis and identified 28 shared targets between HED and ferroptosis (Figure 4A). PPI network analysis of these shared targets was performed using the STRING database. The results showed high network centrality (average node rank = 10.1) and substantial functional correlations (p‐value < 1.0e−16) (Figure 4B). This PPI network was then analyzed topologically using Cytoscape (v3.9.1). By applying the cytoHubba plugin, we found the top 15 hub targets according to the Degree methodology, with the most prevalent targets being TNF, IL6, IL1B, HMOX1, PTGS2, HMGCR, NFE2L2, PPARA, NFKB1, GPX4, ESR1, NR1H4, SLC7A11, G6PD, and TFRC (Figure 4C). Gene Ontology (GO) analysis of the 28 shared targets demonstrated significant enrichment in biological processes (BP) related to ferroptosis regulation, cellular responses to lipopolysaccharide, intracellular iron homeostasis, and iron ion transport. For cellular components (CC), the targets were predominantly localized to protein‐containing complexes, cytoplasmic compartments, and nucleoplasmic structures (Figure 4D,E). Molecular function (MF) analysis revealed significant enrichment of the targets in transcriptional co‐activator binding, nuclear receptor activity, and iron ion binding (Figure 4F). Collectively, these 28 common targets likely mediate the regulatory effects of HED on SOR sensitivity.

### 3.5 HED Suppressed SLC7A11 Expression in the SOR Treated HCC Cells

**FIGURE 5:**
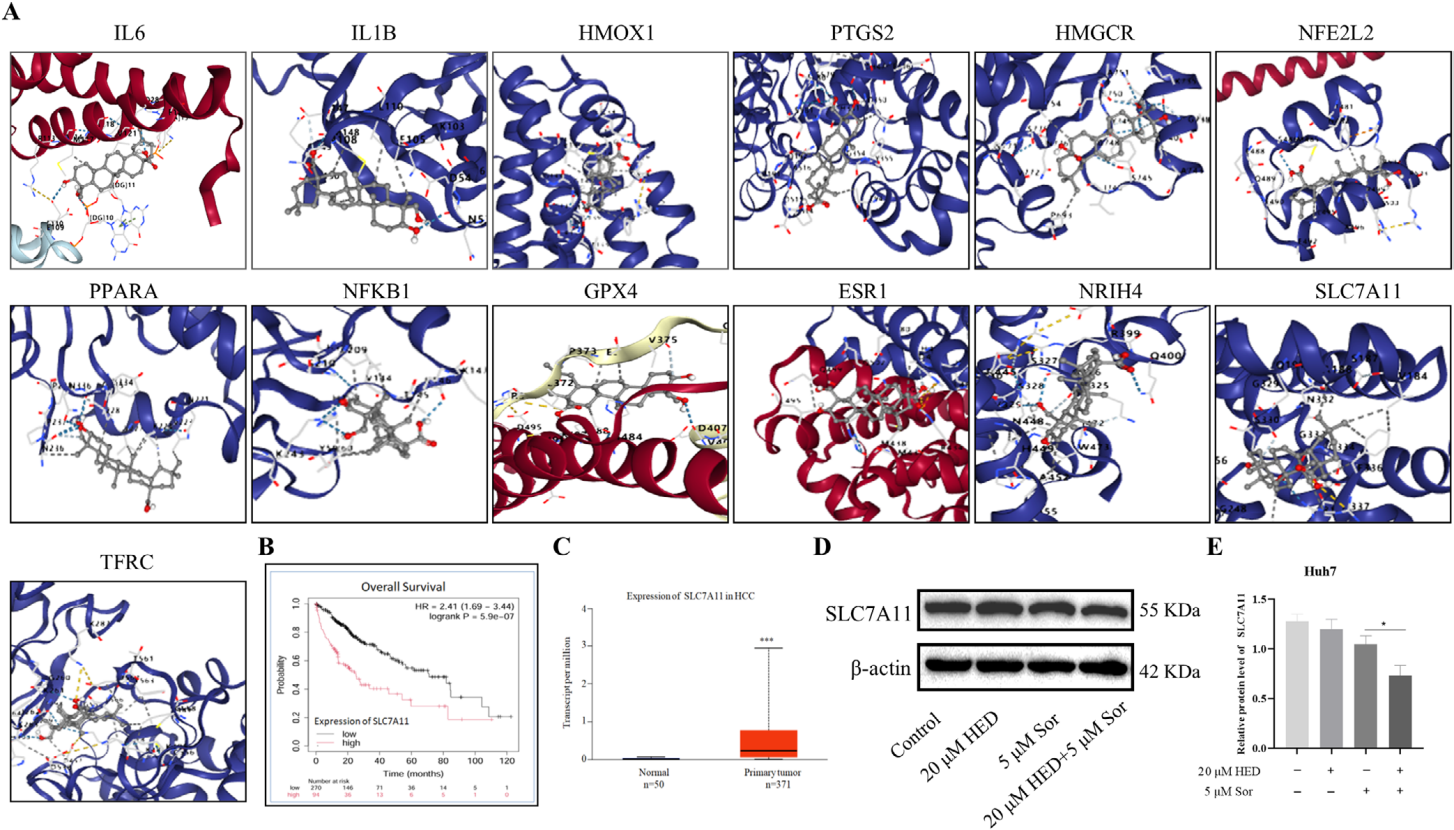
HED suppressed SLC7A11 expression in the SOR treated HCC cells. (A) Molecular docking was used to predict interactions between HED and the top 15 target proteins. (B, C) Online databases were used to analyze the expression levels and overall survival rates of SLC7A11 in HCC, and survival analysis was conducted using the Kaplan–Meier method. (D, E) With the administration of the expression levels of SLC7A11 were examined following HED and SOR co‐treatment. Data were presented as means ± SD (*n* = 3). *p < 0.05; ***p < 0.001, versus 5 μM Sor alone.

To analyze the binding interactions between HED and the 15 primary hub targets identified through network analysis, simulations of molecular docking were performed. As shown in Figure 5A, HED exhibited strong binding affinities for multiple targets implicated in ferroptosis regulation, including IL6, IL1B, HMOX1, PTGS2, HMGCR, NFE2L2, PPARA, NFKB1, GPX4, ESR1, NR1H4, SLC7A11, and TFRC. Notably, HED displayed the highest binding affinity for SLC7A11 (binding energy: −9.2 kcal/mol), a key regulator of lysosomal H^+^ transport and ferroptosis suppression. Analysis using Oncomine online tools revealed that elevated SLC7A11 levels were present in primary HCC tissues (Figure 5B), and they were associated with reduced overall survival by Kaplan–Meier analysis (Figure 5C). Subsequent experimental validation in Huh7 cells revealed that co‐treatment with HED and SOR significantly suppressed SLC7A11 protein expression compared to SOR monotherapy (Figure 5D,E). These findings demonstrated that HED alleviated SOR resistance partially through direct targeting SLC7A11 and modulation of ferroptosis.

### 3.6 Knockdown of SLC7A11 Recapitulated the Sensitizing Effect of HED on Sorafenib

**FIGURE 6:**
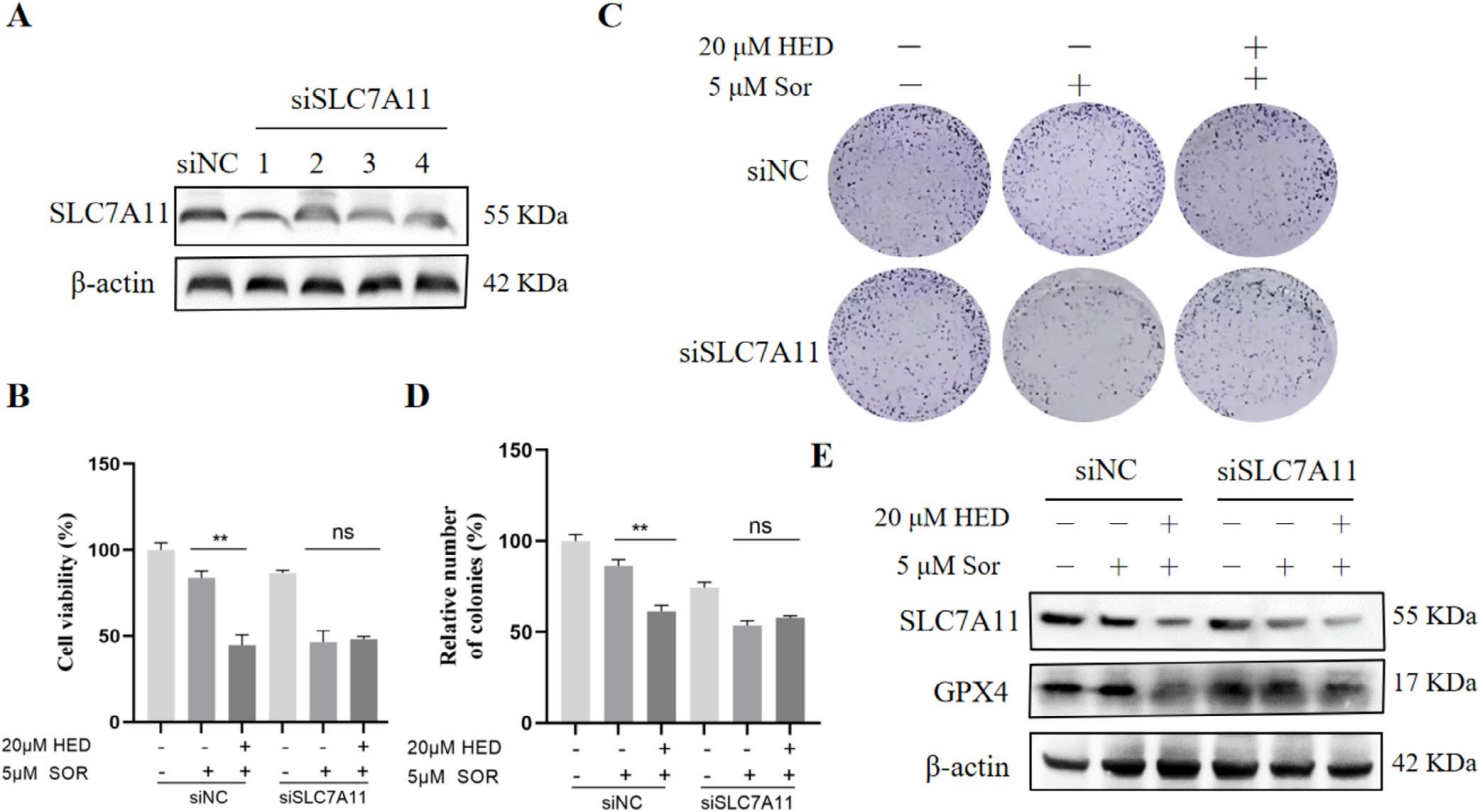
Knockdown of SLC7A11 recapitulated the effect of HED on Sorafenib‐induced ferroptosis. (A) SLC7A11 protein was measured in the SLC7A11 siRNA‐transfected Huh7 cells by western blot. GAPDH was used as the loading control. (B) Cell viability was examined by CCK‐8 assay under the indicated treatments. (C) The protein levels of GPX4 and SLC7A11 were examined by western blot under the indicated conditions. (D, E) Colony formation was examined in control and SLC7A11‐knockdown cells treated with HED and/or sorafenib. Data were presented as means ± SD (*n* = 3). **p < 0.01, versus 5 μM Sor alone.

**FIGURE 7:**
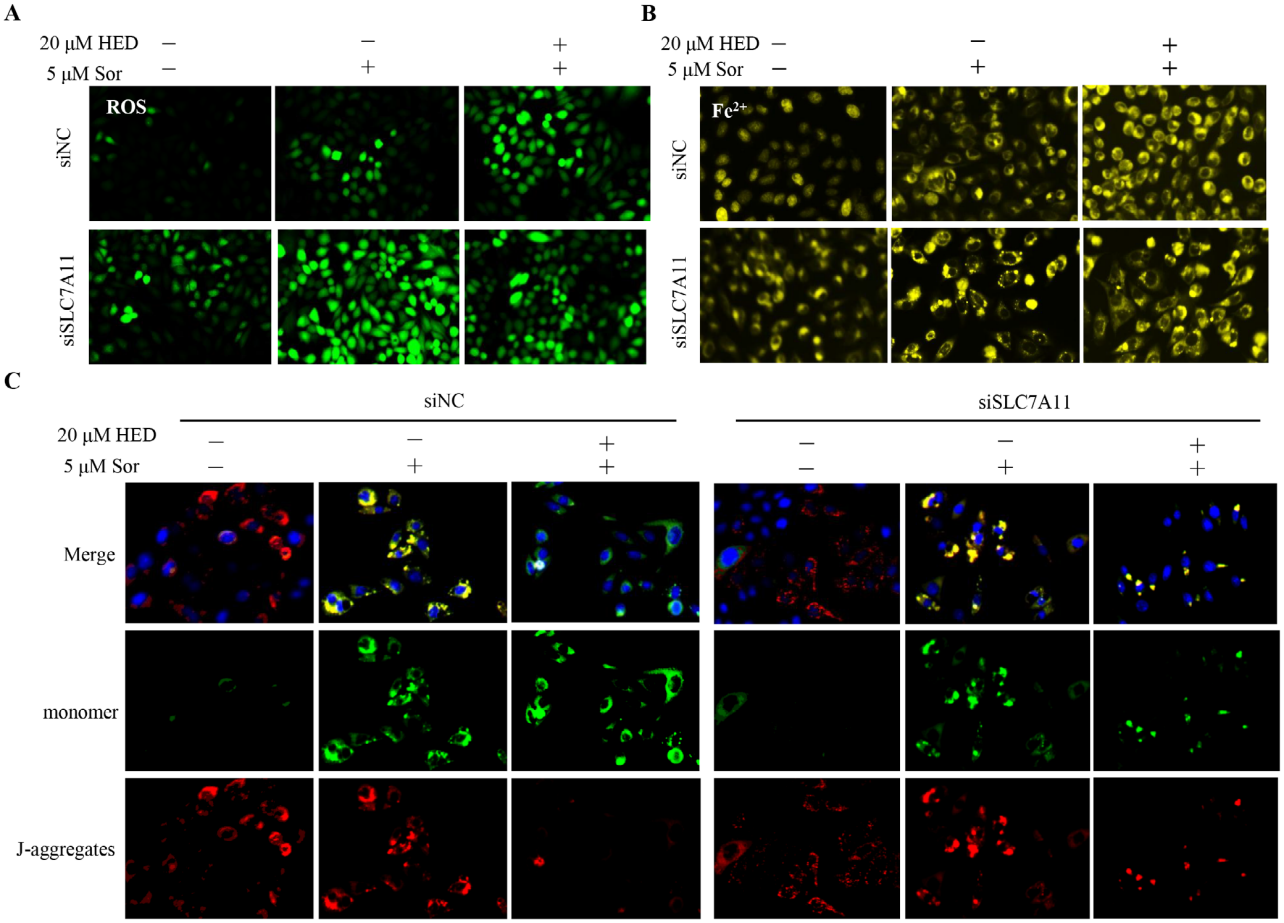
SLC7A11 knockout recapitulates the effect of HED on sorafenib‐induced ferroptosis. (A) Representative fluorescence images and quantification of C11‐BODIPY staining showing lipid ROS levels. (B) FerroOrange staining indicating intracellular Fe^2+^ levels. (C) JC‐1 staining demonstrating changes in mitochondrial membrane potential.

To determine whether SLC7A11 is required for the synergistic effect of HED and sorafenib, we performed siRNA‐mediated knockdown of SLC7A11 in Huh7 cells. Knockdown efficiency was confirmed by Western blotting, and a more than 70% reduction in SLC7A11 protein expression compared with negative control (NC) siRNA‐transfected cells (Figure 6A). Notably, SLC7A11 knockdown alone did not significantly affect cell viability in the absence of sorafenib. However, upon sorafenib treatment, SLC7A11 knockdown substantially reduced cell viability compared with NC siRNA‐transfected cells, as determined by CCK‐8 assay (Figure 6B). SLC7A11 knockdown combined with sorafenib induced cell death to an extent comparable to that observed in NC siRNA‐transfected cells treated with HED and sorafenib (Figure 6C,D), indicating that SLC7A11 knockdown recapitulates the sensitizing effect of HED. Additionally, this combination reduced GPX4 protein expression (Figure 6E). Consistent with these findings, SLC7A11 knockdown significantly increased lipid reactive oxygen species (ROS) levels, as measured by C11‐BODIPY staining (Figure 7A), and elevated intracellular ferrous iron (Fe^2+^) levels, as detected by FerroOrange probe (Figure 7B). SLC7A11 knockdown combined with sorafenib significantly decreased the red/green JC‐1 fluorescence ratio, indicating mitochondrial membrane potential depolarization (Figure 7C). Collectively, these results demonstrate that SLC7A11 knockdown phenocopies the effect of HED treatment, indicating that inhibition of SLC7A11 is sufficient to enhance sorafenib sensitivity through ferroptosis induction.

### 3.7 Overexpression of SLC7A11 Abrogates the Synergistic Antitumor Effect of HED and Sorafenib

**FIGURE 8:**
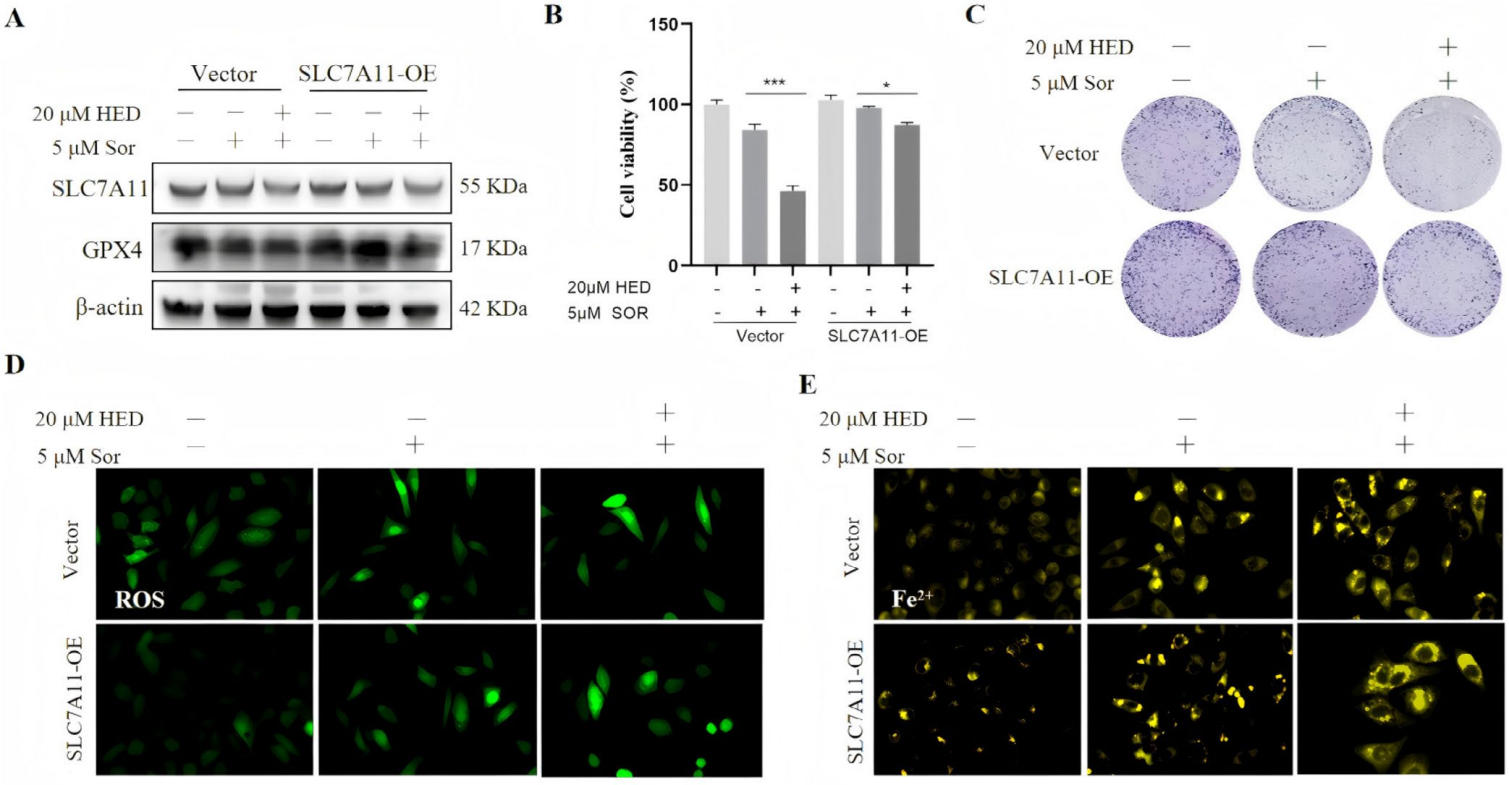
Overexpression of SLC7A11 abrogates the synergistic effect of HED and sorafenib. (A) The expression of SLC7A11 and GPX4 was validated by western blot in Huh7 cells. (B) Cell viabilities were examined by CCK‐8 assay in the SLC7A11‐overexpressing cells with co‐ or alone treatment. (C) Colony formations in the SLC7A11‐overexpressing cells were measured under the indicated treatment. (D) C11‐BODIPY staining for lipid ROS detection. (E) FerroOrange staining for the detection of intracellular Fe^2+^. Data were presented as means ± SD (*n* = 3). *p < 0.05; ***p < 0.001, versus 5 μM Sor alone.

On the other hand, we conducted the gain of function study with the generated SLC7A11‐overexpressing stable Huh7 cells. Successful overexpression was confirmed by Western blotting, and the results showed a marked increase in SLC7A11 protein levels compared with empty vector (Vector) control cells (Figure S2). SLC7A11 overexpression also restored GPX4 protein expression (Figure 8A). Notably, SLC7A11 overexpression significantly attenuated the synergistic cytotoxicity of HED and sorafenib. CCK‐8 assays revealed that SLC7A11‐overexpressing cells exhibited markedly higher cell viability upon HED and sorafenib co‐treatment when compared with Vector control cells (Figure 8B). Similarly, colony formation assays confirmed that the inhibitory effect of the combination treatment was substantially diminished by SLC7A11 overexpression (Figure 8C). Mechanistically, SLC7A11 overexpression suppressed the ferroptosis induced by HED and sorafenib. Compared with Vector control cells, SLC7A11‐overexpressing cells showed significantly lower lipid ROS accumulation (Figure 8D) and reduced ferrous iron levels (Figure 8E) upon combined treatment. Collectively, these results demonstrate that SLC7A11 overexpression abolishes the synergistic effect of HED and sorafenib, further confirming the involvement of ferroptosis.

## 4 Discussion

Sorafenib (SOR), a multikinase inhibitor targeting VEGF receptors, PDGFR‐β, and RAF/MEK/ERK pathways, has demonstrated significant survival benefits in pivotal clinical trials for advanced HCC (Abou‐Alfa 2009). The SHARP trial, known as the Sorafenib HCC Assessment Randomized Protocol, was groundbreaking in determining that SOR is the initial systemic treatment for HCC that has not undergone surgical removal, gaining approval from the FDA (Llovet et al. 2021). Nonetheless, the rapid development of both primary and acquired drug resistance significantly undermines its clinical effectiveness. Addressing SOR resistance and increasing its therapeutic sensitivity presents a promising approach to enhance clinical outcomes for individuals with advanced HCC (Tang et al. 2020).

Traditional Chinese medicine (TCM) exhibits promising potential in reversing SOR resistance and serving as a synergistic adjuvant for advanced HCC therapy, as evidenced by its multi‐target modulation of ferroptosis, metabolic reprogramming, and tumor microenvironment crosstalk (Yang et al. 2024, 2021; Lu, Zhu, et al. 2024). Hederagenin (HED), a bioactive pentacyclic triterpenoid derived from traditional Chinese medicinal herbs such as *Hedera helix* and *Aralia chinensis* , demonstrates broad‐spectrum antitumor activity through multi‐target modulation of oncogenic signaling pathways (Kim et al. 2017). Accumulating evidence underscores its potential to overcome chemoresistance in various cancers. For example, HED counteracts oxaliplatin resistance in gastric cancer by blocking the PI3K/AKT/mTOR pathway, a crucial factor in therapy evasion (Tang et al. 2024). In this study, we found that HED serves as a powerful chemosensitizer for SOR, which is the primary tyrosine kinase inhibitor employed in treating advanced HCC. Notably, HED enhanced the effectiveness of SOR, leading to a reduction of its IC50 in HCC cell lines by more than 50%. This synergistic effect can be understood through HED's ability to augment SOR‐induced ferroptosis via the dual regulation of iron metabolism and lipid peroxidation pathways.

It has been reported that sorafenib can facilitate ferroptosis in cancer cells, and preventing this process is vital for sorafenib resistance in HCC cells (Gao et al. 2021). With SOR treatment, HED was found to significantly raise intracellular concentrations of ROS, Fe^2+^, and MDA, whereas lowering decreased GSH levels in HCC cells. Conversely, the rescue study used Fer‐1, a ferroptosis inhibitor, which showed that ferroptosis suppression greatly reduced the cell mortality brought on by the combination of HED and SOR therapy. These data indicate that HED plays a crucial role in improving sorafenib sensitivity by triggering ferroptosis in HCC cells.

To explore the molecular pathways by which HED can transcend SOR resistance, we performed a bioinformatics analysis to determine the shared targets of HED and ferroptosis. A total of 28 shared targets were identified, of which the top 15 targets including TNF, IL6, IL1B, HMOX1, PTGS2, HMGCR, NFE2L2, PPARA, NFKB1, GPX4, ESR1, NR1H4, SLC7A11, G6PD, and TFRC are critically involved in ferroptosis regulation, iron homeostasis, and transport pathways. Notably, HMGCR, the mevalonate pathway's rate‐limiting enzyme, drives ferroptosis resistance via GSH/GPX4 and FSP1/CoQ10 axes by producing mevalonate‐derived metabolites like IPP and CoQ10 (Juarez and Fruman 2021; Gong et al. 2019), while ESR1 inhibits ferroptosis through NEDD4L/CD71‐mediated iron export in breast cancer (Liu et al. 2022), and G6PD overexpression in HCC suppresses ferroptosis via NADPH‐dependent cytochrome oxidoreductase activity (Cao et al. 2021). These findings align with Elabela's modulation of IL‐6/STAT3/GPX4 to attenuate iron overload and lipid peroxidation (Zhang, Tang, et al. 2022), collectively highlighting HED's multi‐target potential to overcome SOR resistance by disrupting redox and iron metabolic networks.

To further validate the binding affinity between HED and the top 15 predicted targets, we conducted a molecular docking analysis. The findings demonstrated a strong interaction capability among these targets, with the most significant interaction identified for SLC7A11 (binding energy: −7.9 kcal/mol), which is comparable to triterpenoids such as oleanolic acid in terms of viral protease inhibition. Commonly referred to as xCT, SLC7A11 functions as the light‐chain subunit of the cystine/glutamate antiporter system xC−, permitting the import of external cystine to be exchanged for glutamate from inside the cell. Notably, SLC7A11 is often upregulated in various tumors, providing resistance against ferroptosis by maintaining GSH/GPX4 activity. Prior research has confirmed that ferroptosis driven by SLC7A11 is a mediating factor in the anti‐tumor properties of SOR in hepatocellular carcinoma, gastric cancer, and colon cancer (Huang et al. 2021; Li et al. 2022). This research revealed that SLC7A11 was notably increased in HCC tissues and strongly correlated with poor survival rates among HCC patients. Validation experiments in Huh7 cells demonstrated a decrease in SLC7A11 expression in cells co‐treated with HED and SOR, suggesting that SLC7A11 could be a key target of HED. The loss of and gain of function studies further verified that SLC7A11 really served as a target of HED in the SOR‐induced ferroptosis. However, this study is not without its limitations. Due to constraints related to budget and time, the specific molecular mechanisms that underpin HED's role in SOR sensitivity and its functional effects in vivo have not yet been investigated. Additional experiments will be conducted to confirm the functional and mechanistic roles of HED in overcoming SOR resistance in future studies.

In summary, our results indicate that HED enhances the anti‐tumor activity of SOR in HCC cells via downregulating SLC7A11 expression and inducing ferroptosis. These findings suggest that HED may act as an effective strategy to overcome SOR resistance and improve its efficacy in refractory HCC.

## Author Contributions

**Jian‐yu Zhang:** data curation, visualization. **Jin‐Fang Zhang:** conceptualization, project administration, funding acquisition, writing – review and editing. **Fu‐zhen Pan:** funding acquisition, methodology, software, formal analysis. **Ya‐ge Zhang:** methodology, data curation, resources, writing – review and editing, funding acquisition. **Yu‐wan Zhou:** methodology, data curation, investigation, validation. **Hai‐mei Jiang:** methodology, data curation, writing – original draft, investigation.

## Funding

This work was supported by National Natural Science Foundation of China (82772526, 82575166), Guangzhou University of Chinese Medicine (GZYFT2024G11), Science, Technology and Innovation Commission of Shenzhen Municipality (JCYJ20250604190524030, JCYJ20250604191035045), and Sanming project of medicine in Shenzhen (SZZYSM202311011).

## Ethics Statement

The authors have nothing to report.

## Conflicts of Interest

The authors declare no conflicts of interest.

## Supporting information


**Figure S1:** (A–D) Following treatment with 20 μM HED, cell viability was evaluated at 48 and 72 h, and the IC50 value of SOR was determined in Huh7 and HepG2 cells. Data were presented as means ± SD (*n* = 3). *p < 0.05; **p < 0.01; ***p < 0.001, versus 5 μM Sor alone.
**Figure S2:** The expression levels of SLC7A11 in Huh7 cells were increased with overexpressing SLC7A11.

**Table S1:** The sequences of siRNA used in this study.

## Data Availability

The data that support the findings of this study are available from the corresponding author upon reasonable request.
