# Supplementary material for: Hederagenin Promotes Sorafenib Sensitivity in Hepatocellular Carcinoma Through Suppressing SLC7A11 Expression and Inducing Ferroptosis
Source: Food Sci Nutr. 2026 May 22;14(5):e71873. doi: 10.1002/fsn3.71873 (PMC13240546; doi:10.1002/fsn3.71873)
Supplement: Supplementary file 1 — Figure S1: (A–D) Following treatment with 20 μM HED, cell viability was evaluated at 48 and 72 h, and the IC50 value of SOR was determined in Huh7 and HepG2 cells. Data were presented as means ± SD (n = 3). *p < 0.05; **p < 0.01; ***p < 0.001, versus 5 μM Sor alone. Figure S2: The expression levels of SLC7A11 in Huh7 cells were increased with overexpressing SLC7A11. [file FSN3-14-e71873-s002.docx]

**
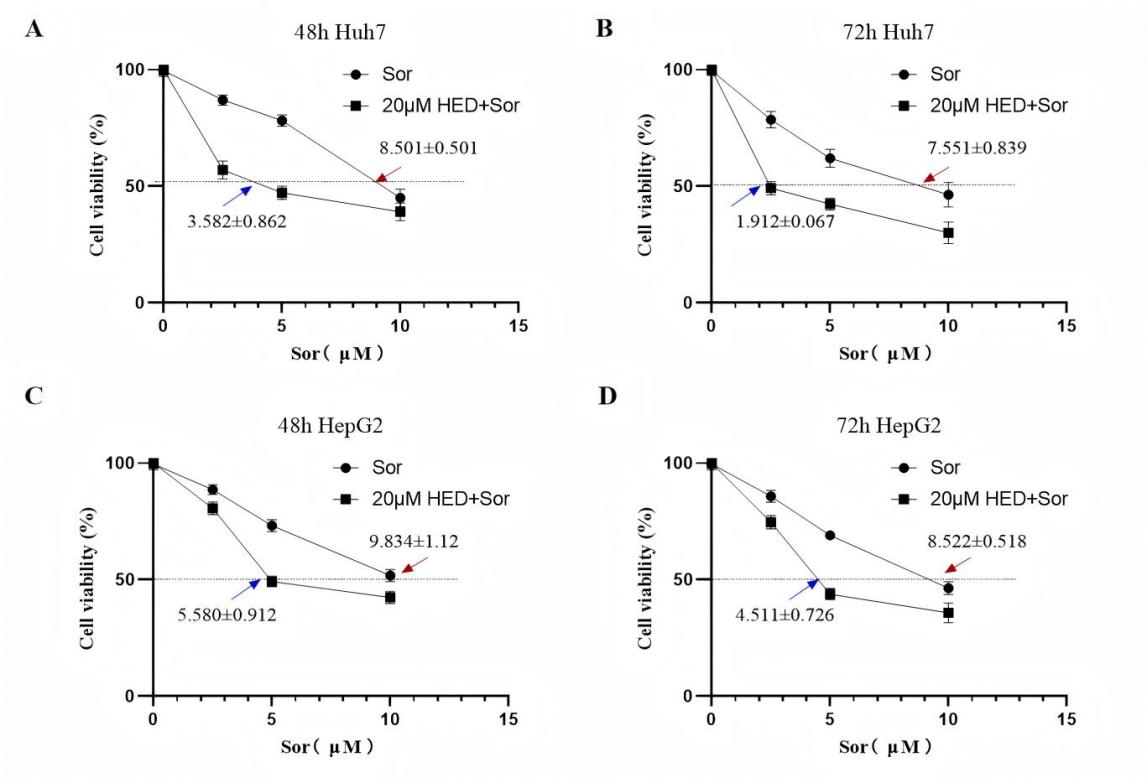
**

**Figure S1.** A-D, Following treatment with 20 μM HED, cell viability was evaluated at 48 h and 72 h, and the IC50 value of SOR was determined in Huh7 and HepG2 cells. Data were presented as means ± SD (n=3). *, p<0.05; **, p<0.01; ***, p<0.001, versus 5µM Sor alone.

**
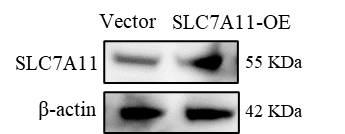
**

**Figure S2.** The expression levels of SLC7A11 in Huh7 cells were increased with overexpressing SLC7A11.
