# Supplementary material for: Hederagenin Promotes Sorafenib Sensitivity in Hepatocellular Carcinoma Through Suppressing SLC7A11 Expression and Inducing Ferroptosis
Source: Food Sci Nutr. 2026 May 22;14(5):e71873. doi: 10.1002/fsn3.71873 (PMC13240546; doi:10.1002/fsn3.71873)
Supplement: Supplementary file 2 — Table S1: The sequences of siRNA used in this study. [file FSN3-14-e71873-s001.docx]

**Table S1.** The sequences of siRNA used in this study

| **Gene name** | **Target sequence** |
| --- | --- |
| SLC7A11-siRNA#1 | GGGUGGAACUCCUCAUAAUTT AUUAUGAGGAGUUCCACCCTT |
| SLC7A11-siRNA#2 | CACCCUUUGACAAUGAUAATT  UUAUCAUUGUCAAAGGGUGTT |
| SLC7A11-siRNA#3 | CUGCGUAUUAUCUCUUUAUTT  AUAAAGAGAUAAUACGCAGTT |
| SLC7A11-siRNA#4 | GGGCUGAUUUAUCUUCGAUTT  AUCGAAGAUAAAUCAGCCCTT |
| NC-siRNA | UUCUCCGAACGUGUCACGUTT  ACGUGACACGUUCGGAGAATT |
